# Erdheim–Chester Disease Manifesting Without Long Bone Involvement

**DOI:** 10.1002/rcr2.70237

**Published:** 2025-06-10

**Authors:** Dhiran Sivasubramanian, Karthick Balasubramanian, Mohamed Raghib Hussain Mohamed Kalifa, Sathwik Sanil, Smrti Aravind, Nithish Nanda Palanisamy, Virushnee Senthilkumar

**Affiliations:** ^1^ Department of Cardiology Children's Hospital of Philadelphia Philadelphia Pennsylvania USA; ^2^ Department of Critical Care Medicine Christian Medical College Vellore India; ^3^ Department of Cardiology MGM Healthcare Chennai India; ^4^ Institute of Oncology, Sri Ramakrishna Hospital Coimbatore India; ^5^ Department of General Medicine Coimbatore Medical College Coimbatore India

**Keywords:** CD163‐positive histiocytes, erdheim–chester disease, foamy histiocytes, non‐langerhans cell histiocytosis, skeletal sparing

## Abstract

Erdheim–Chester Disease (ECD) is an extremely rare, non‐Langerhans cell histiocytosis characterised by the proliferation of foamy histiocytes infiltrating various organs. It almost always presents with osteosclerosis of the long bones, making our case atypical due to the absence of skeletal involvement despite widespread infiltration of other organs.

## Graphical abstract

We present an atypical case of Erdheim–Chester Disease (ECD) due to the absence of skeletal involvement despite widespread infiltration of other organs.

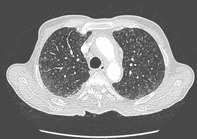

A 59‐year‐old man with no prior respiratory illness presented with progressive breathlessness over 9 months, which worsened in the past 15 days. He also reported orthopnea, bilateral pedal oedema, and a chronic mucoid cough. His medical history included type 2 diabetes mellitus, systemic hypertension, chronic kidney disease (diabetic nephropathy), and coronary artery disease. He had no history of smoking or tuberculosis exposure. Over the past 8 months, he experienced two unexplained syncopal episodes and noted progressive facial skin lesions.

On examination, he had facial xanthelasmas, bilateral leg oedema, and reduced breath sounds on the right. Vitals were stable with SpO_2_ at 94%. Lab tests revealed microcytic anaemia, elevated creatinine (2 mg/dL), and urea (72 mg/dL). Chest X‐ray showed right mid‐ and lower‐zone consolidation and a blunted costophrenic angle (Figure 1). HRCT and CECT revealed bilateral interlobular nodules with a branching pattern, pericardial effusion, retrobulbar soft tissue lesions, perinephric thickening, and para‐aortic soft tissue enhancement (Figures 2 and 3). PET‐CT confirmed these findings, with no long bone involvement.

**FIGURE 1:**
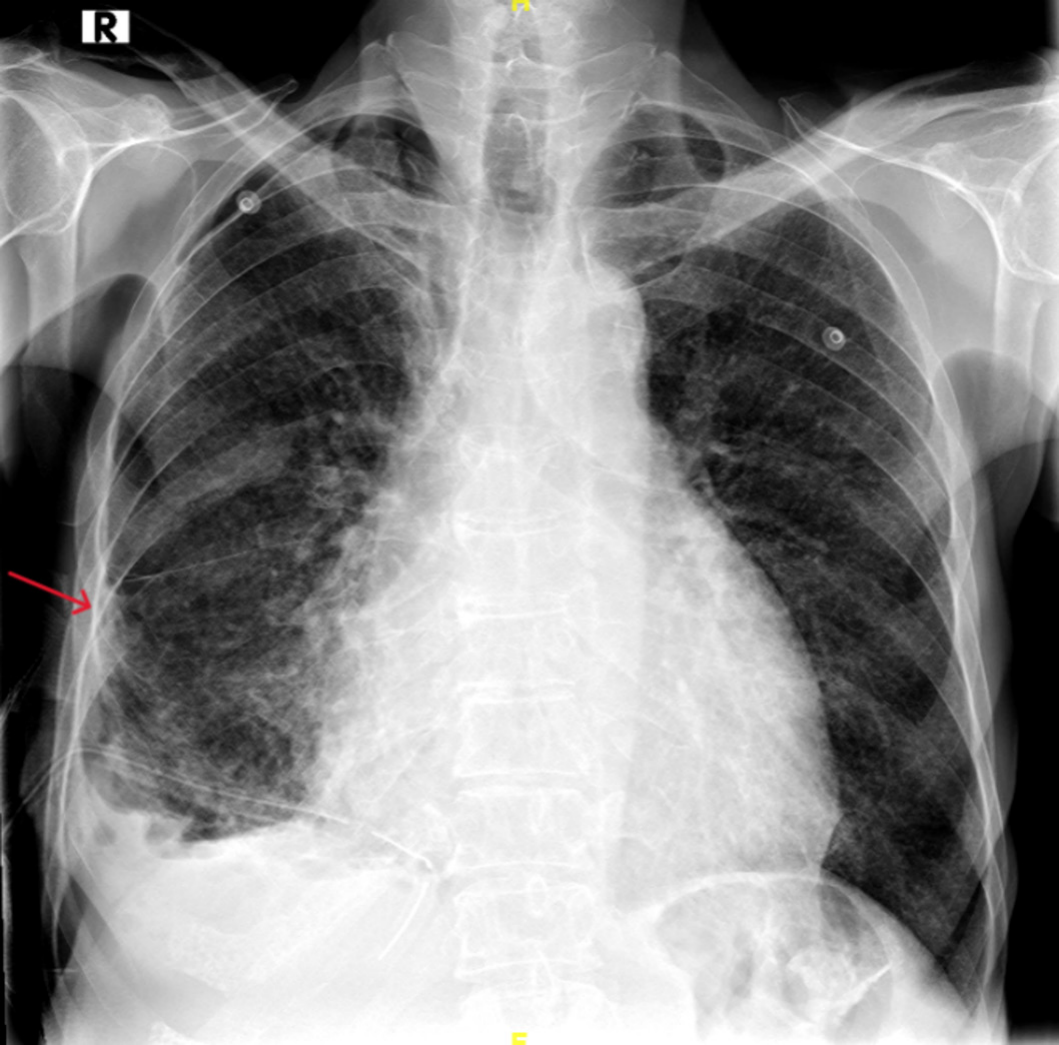
Plain chest radiograph (X‐Ray) post chest tube insertion showing right mid and lower zone consolidation and a blunted costophrenic angle.

**FIGURE 2:**
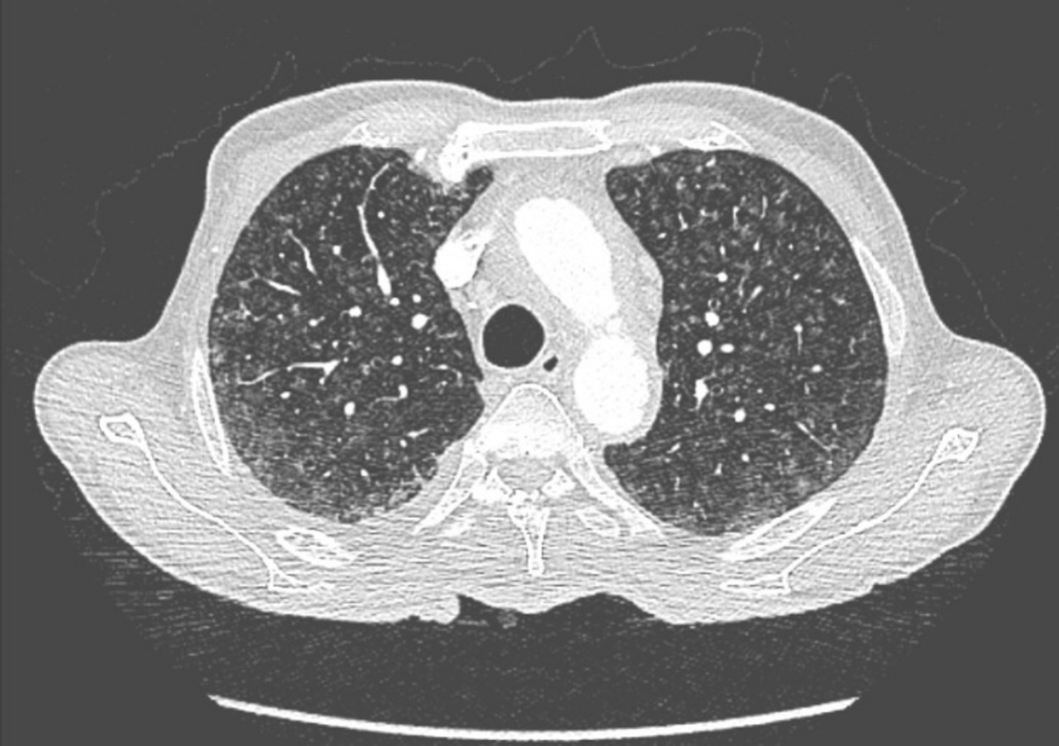
High‐resolution computed tomography (HRCT) of the chest showing bilateral milliary pattern with centrilobular nodules.

**FIGURE 3:**
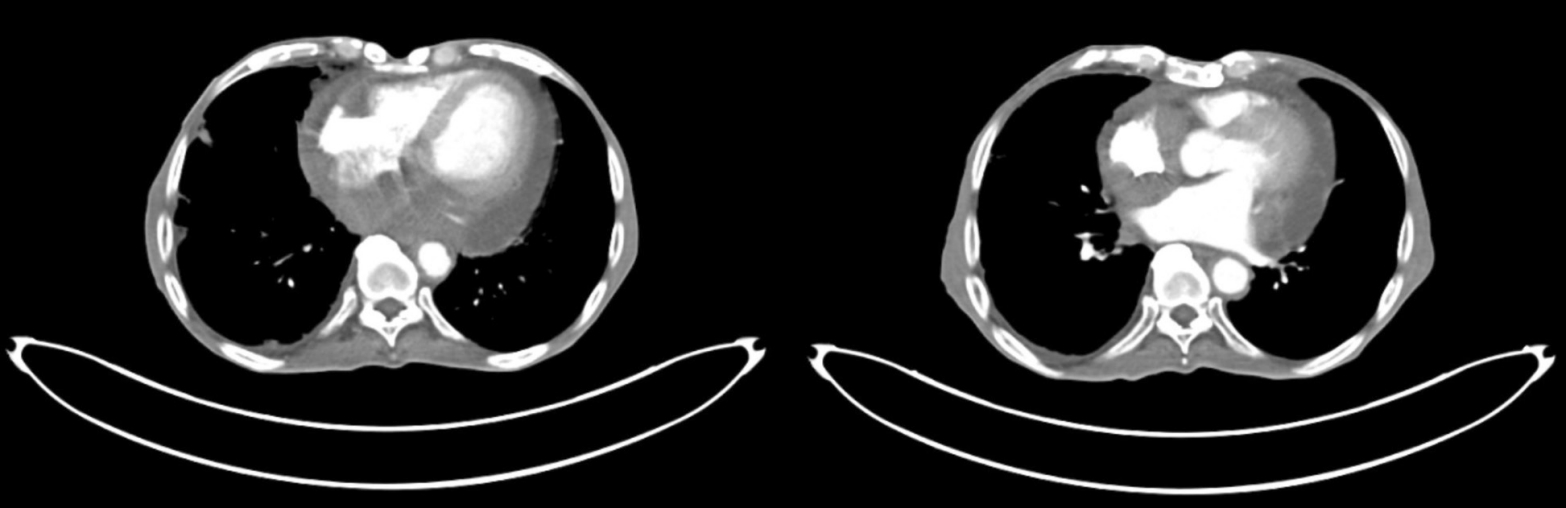
Contrast‐enhanced computed tomography (CECT) showing paracardiac soft tissue enhancement surrounding both the atria, basal parts of ventricles, atrio‐ventricular grooves and medial aspect of ascending aorta.

A diagnosis of ECD was confirmed by skin and transbronchial biopsies showing foamy histiocytes with Touton giant cells, positive for CD163 and negative for CD1a, S100 and BRAFV600E [1]. Treatment with interferon alpha was initiated.

ECD is a rare non‐Langerhans histiocytosis, typically causing osteosclerosis of the long bones [2]. This case is atypical due to the absence of skeletal involvement despite widespread multi‐organ involvement, highlighting the disease's protean manifestations and the need for a multidisciplinary diagnostic approach.

## Author Contributions

**Dhiran Sivasubramanian:** conceptualisation, data curation, project administration, supervision, original draft writing, review, and editing. **Karthick Balasubramanian:** patient evaluation, conceptualisation, investigation, review, and editing. **Mohamed Raghib Hussain Mohamed Kalifa:** conceptualisation, data curation, investigation, and project administration. **Sathwik Sanil:** conceptualisation, investigation, project administration. **Smrti Aravind:** visualisation, writing – review, and editing. **Nithish Nanda Palanisamy:** image selection, data curation. **Virushnee Senthilkumar:** visualisation, writing – review, and editing.

## Ethics Statement

All the data of this study were taken from the medical records of the patient. This report does not contain any personal information that could lead to the identification of the patient.

## Consent

The authors declare that written informed consent was obtained for the publication of this manuscript and accompanying images using the consent form provided by the Journal.

## Conflicts of Interest

The authors declare no conflicts of interest.

## Data Availability

Data sharing not applicable to this article as no datasets were generated or analysed during the current study.
